# ADULT CHILD PROXIMITY AND REPARTNERING AMONG PARENTS AFTER GRAY DIVORCE

**DOI:** 10.1093/geroni/igac059.156

**Published:** 2022-12-20

**Authors:** Susan Brown, I-Fen Lin, Kagan Mellencamp

**Affiliations:** Bowling Green State University, Bowling Green, Ohio, United States; Bowling Green State University, Bowling Green, Ohio, United States; Bowling Green State University, Bowling Green, Ohio, United States

## Abstract

The rise in gray divorce is spurring growth in repartnering, which occurs more frequently after divorce than widowhood. Roughly 40% of men and 25% of women form a co-residential union following gray divorce. Drawing on the 1998-2014 Health and Retirement Study, we assessed whether parent-adult child proximity (living within 10 miles) was associated with men’s and women’s repartnering after gray divorce. Men’s relationships with their children tend to erode following gray divorce, and thus we anticipated that the association between proximity and repartnering was negligible. In contrast, women’s ties to adult children are largely resilient following gray divorce, which led us to expect that having a child nearby reduced chances of repartnering. Our analyses revealed that both men and women with a proximate child less often repartnered than those with no children nearby. Repartnering seemingly functions as an alternative means to “doing family” for those lacking nearby children.

